# Trust by design: the effects of personal interest vs. social responsibility frame on policy acceptance and patient trust in China’s DRG reform

**DOI:** 10.1186/s12913-026-14681-1

**Published:** 2026-05-11

**Authors:** Zichun Leng, Yang Chen

**Affiliations:** School of Journalism and Communication, Renmin University of China, Zhongguancun Avenue 59, Beijing, 100872 China

**Keywords:** DRG, Frame, Patient trust, Policy acceptance

## Abstract

**Background:**

Diagnosis Related Groups (DRG) payment reform has been widely adopted internationally to improve efficiency and contain healthcare costs. In recent years, China has accelerated the implementation of DRG and Diagnosis-Intervention Packet (DIP) payment systems, with more than 90% of pooled regions adopting these mechanisms by the end of 2023. While such reforms have standardized service delivery and reduced patient expenses, public awareness and understanding remain limited, raising concerns about their potential impact on patient trust. This study examines how personal interest versus social responsibility framing influences patients’ trust in the healthcare system and the acceptance of DRG payment reform.

**Methods:**

A survey experiment was conducted among obstetric patients of different due dates (*N* = 229) in a region where DRG reforms have been implemented in China, assessing responses to 2 types of messages designed to increase awareness and acceptance of DRG policies. Independent-samples t-tests were used to compare groups of different frames. Mediation effects of patient trust and potential moderating effects of temporal distance were tested using a structural equation model, and regression models were employed to examine predictors of specific trust.

**Results:**

Compared to the social responsibility frame, participants exposed to the personal interest frame reported higher levels of general patient trust, with the difference driven primarily by trust in the healthcare system rather than trust in the medical profession. Structural equation modeling indicated a significant indirect association between framing condition and policy acceptance through general patient trust. In addition, general patient trust was positively associated with specific trust. Temporal distance and its interaction with framing were not significantly associated with policy acceptance.

**Conclusion:**

Framing policies in terms of personal benefits is more effective than emphasizing social responsibility in enhancing general patient trust. Even when such frames do not directly increase policy acceptance, general patient trust emerged as an important psychological pathway connecting message framing to reform evaluation. Against the backdrop of low public awareness of DRG reform, these findings underscore the importance of clearer and more accessible policy communication.

**Clinical trial number:**

Not applicable.

**Supplementary Information:**

The online version contains supplementary material available at 10.1186/s12913-026-14681-1.

## Introduction

Diagnosis Related Groups (DRG) payment is a prospective bundled payment method in which hospitalized patients are classified into groups according to similar clinical features and resource consumption, with medical cost standards set for each group [1]. Under this payment system, pharmaceuticals, medical supplies, and diagnostic tests are no longer sources of hospital revenue, but rather costs associated with disease treatment. Originating in the United States in the 1980s, DRG-based systems have since been implemented across various countries, including Australia, Germany, France, the United Kingdom, Thailand, Singapore, and China recently [2], achieving controversial yet mainly positive outcomes in improving healthcare efficiency [3–4]. China has been exploring the Pricing and Payment Regulation on Chinese Diagnosis Related Groups (C-DRG) since 2010, and accelerated its efforts in the past two years [5–6]. By the end of 2023, more than 90% of pooled regions across China had implemented DRG or Diagnosis-Intervention Packet (DIP)-based payment mechanisms [7].

Despite their growing prevalence, DRG reforms are often introduced and implemented with limited public engagement. While such reforms may reduce over-commercialization and improve hospital efficiency, they can also provoke concerns about compromised care quality or financial motivations behind medical decisions. These concerns may erode public trust in healthcare systems, especially when reforms are poorly communicated to patients.

Prior research has primarily focused on provider behaviors and institutional outcomes of DRG adoption [8, 9], with much less attention given to how patients perceive and respond to these policy changes. A critical yet underexplored question is how message framing, particularly emphasizing personal versus social benefits, can influence patients’ acceptance of DRG reforms and their trust in the healthcare system.

Besides, the impact of framing on patient trust and policy acceptance might be moderated by psychological distance, which shapes how individuals cognitively process and evaluate framed information in relation to themselves.

Using a case from a DRG pilot region in China, the study explores the optimal communication framework for the DRG policy and aims to enhance pathways for increasing patient trust through a survey-based experiment. Under the current dual decision-making model of “government–scientist” dominance in China’s health policy domain, the lack of citizen participation has widened the information asymmetry in doctor–patient relationships [10]. Thus, China provides a valuable setting to examine how communication framing might help mitigate information asymmetries and foster greater trust in healthcare reforms.

## Literature review

### Framing the benefits

Tversky and Kahneman demonstrated that equivalent information, when presented differently, can lead to distinct choices [11]. Such “framing effects” occur when small changes in presentation produce significant shifts in opinion [12]. Prior research has identified effective frames across various domains; for example, gain-framed messages better promote disease prevention, while loss-framed messages are more effective in encouraging health screenings [13]. However, no universal theory predicts which frame is optimal for a given issue [12].

Communication studies show that media and news frames also significantly influence public perceptions of policies [14, 15]. For instance, public support for a public insurance option varied from 46.5% to 64.6% depending on survey wording [16], highlighting the pivotal role of framing in shaping policy attitudes.

An analysis of news articles from People’s Daily (http://www.people.com.cn/) using “DRG” as a keyword identified two prominent frames: a “social responsibility” frame, emphasizing societal benefits such as overall cost reduction and resource efficiency, and a “personal interest” frame, focusing on individual gains like reduced personal out-of-pocket expenses and improved patient experience.

Previous studies have shown that frames emphasizing individual outcomes are often more effective in prompting immediate behavior change [12, 17]. In climate change communication, public interest frames foster policy support, whereas personal risk frames evoke stronger emotional and behavioral responses [18]. Similarly, Jacoby found that personal interest frames generated greater support for specific educational policies than broader social benefit frames [19]. It should be noted, however, that framing effects may vary across sociocultural contexts. Building on this evidence, this study assumes:

#### H1

Participants exposed to the personal interest frame will report higher levels of (a) general patient trust, (b) specific patient trust, and (c) policy acceptance compared to those exposed to the social responsibility frame.

### Patient trust

Patient trust is reflected in individuals’ expectations of physicians’ behavior and their willingness to rely on physicians [20]. Due to the life-critical, uncertain, and irreversible nature of medical practice, patient trust is particularly essential [21, 22].

In China, doctor–patient tensions have long been a significant concern. 66% of physicians reported experiencing conflict with patients [23], and malpractice lawsuits rose from 12,734 cases in 2017 to 18,670 in 2020 [24]. Particularly, hospital commercialization and distorted physician income structures have contributed to overtreatment and widespread distrust, following healthcare marketization reforms [25, 26].

Patient trust is shaped by both individual and institutional factors. On the patient side, demographics (e.g., age, gender, education) and subjective perceptions (e.g., perceived health status, perceived stress) are influential [21, 27, 28]. On the physician side, competence, communication skills, professionalism, and situational factors are significant contributors [29]. Beyond interpersonal interactions, institutional arrangements such as health insurance systems and healthcare policies constitute an important structural foundation of patient trust [20, 30]. Drawing on data from the CSS2017 and CSS2023 surveys, Chi further demonstrates that institutional factors exert a stronger influence on doctor–patient trust than cultural factors, highlighting the primacy of systemic conditions in shaping trust perceptions [31].

Correspondingly, patient trust can be conceptualized as two interrelated components: general trust in the healthcare system and the medical profession, and specific trust in individual physicians within concrete clinical encounters [32]. The distinction between general and specific trust has been widely adopted in understanding social trust structures [33]. General trust refers to a broad expectation that institutions and their representatives will act in a reliable and appropriate manner, based on perceived institutional arrangements and shared normative standards [34]. In the healthcare context, general trust is rooted in evaluations of the healthcare system and medical institutions, and is sustained by legal regulations, policy frameworks, professional norms, and shared moral principles. In contrast, specific trust is inherently situational and relational [33]. Through repeated encounters and accumulated experiences, patients form judgments about the trustworthiness of individual doctors. Moreover, existing research suggests that general trust and specific trust are positively associated. Qualitative evidence from Li and Tang further shows that higher levels of general trust in the healthcare system are associated with stronger trust in individual physicians during clinical encounters [32]. However, most studies conceptualize patient trust primarily as interpersonal trust [27–29], focusing on perceptions of physician competence and motivations, while paying less attention to institutional trust in the healthcare system as a whole. Based on these considerations, the following hypothesis is proposed:

#### H2

Greater general patient trust will predict greater specific patient trust.

### Policy acceptance

Policy acceptance refers to the judgments made by target groups regarding public policies that affect their own interests [35, 36]. Enhancing individuals’ positive perceptions of the policy-making process contributes to policy legitimacy and government credibility [36, 37]. Most existing studies on DRG reform focus on healthcare providers, such as physicians’ knowledge, attitudes, practices, and deviant behaviors [8, 38], while research on public policy acceptance remains limited.

Two major perspectives explain the formation of policy acceptance [39]. One views it as a rational process shaped by perceived risks and benefits, aligning with the personal interest frame. The other sees it as a moral process influenced by public moral consciousness, corresponding to the social responsibility frame. In practice, motivations for policy acceptance often combine self-interest and altruism [40]. Also, public judgments of healthcare reforms often prove more nuanced than those assumed by government actors or portrayed in media narratives. Jacobs and Shapiro found public attitudes toward health reform were not merely self-interested or anti-regulation in the US [41]. These findings indicate that framing strategies emphasizing either personal benefits or social responsibility may activate different evaluative pathways in shaping policy acceptance.

The healthcare insurance system represents a crucial institutional factor influencing doctor-patient relationships. A well-functioning healthcare insurance system can foster harmonious doctor-patient relationships and facilitate broader insurance coverage, while high out-of-pocket costs weaken patient trust [42, 43]. Previous research shows that trust in institutions and authorities significantly predicts support for public policies, particularly in health-related contexts [44]. Trust in government and experts has been identified as a key predictor of policy endorsement and compliance during public health interventions [45, 46]. Importantly, declining trust does not merely reflect dissatisfaction with political leaders but also independently reduces support for policy expansion [47]. When patients perceive the healthcare system and its actors as trustworthy, they are more likely to interpret reforms as legitimate and beneficial [48], thereby increasing policy acceptance. Conversely, distrust may lead individuals to interpret reforms as threatening, unfair, or strategically motivated, thus reducing their willingness to endorse policy change. Thus, this study hypothesizes that:

#### H3

(a) general patient trust and (b) specific patient trust will mediate the effect of different framing conditions on policy acceptance.

### Temporal distance

The effectiveness of framing is closely linked to individual cognitive processes, as salient elements highlighted by frames are more likely to capture public attention and be stored in memory. Whether these elements ultimately influence individuals depends on their applicability to the self. Trope introduced construal level theory, defining psychological distance as the perceived gap between the self and an object across four dimensions: time, space, social distance, and hypotheticality. As psychological distance increases, people tend to use more abstract, generalized concepts [49].

When psychological distance is short, individuals evaluate policy risks and benefits more concretely and rationally [50]. In contrast, distant events are judged more through moral norms rather than instrumental reasoning [51]. Moreover, personal interest has a stronger influence on policy preferences when the personal costs and benefits are clear [52].

Therefore, psychological distance may moderate how different frames impact patient trust and policy acceptance. In this study, temporal and hypothetical distances are important for patients’ perceptions of the DRG policy. Given the difficulty in operationalizing hypotheticality, this study uses pregnant women as participants, with childbirth representing a behavior with minimal hypothetical distance. The number of days to the expected due date is used as a measure of temporal distance. Thus, the following hypothesis is proposed:

#### H4

Temporal distance moderates the effects of framing on general patient trust and specific patient trust.

**Fig. 1:**
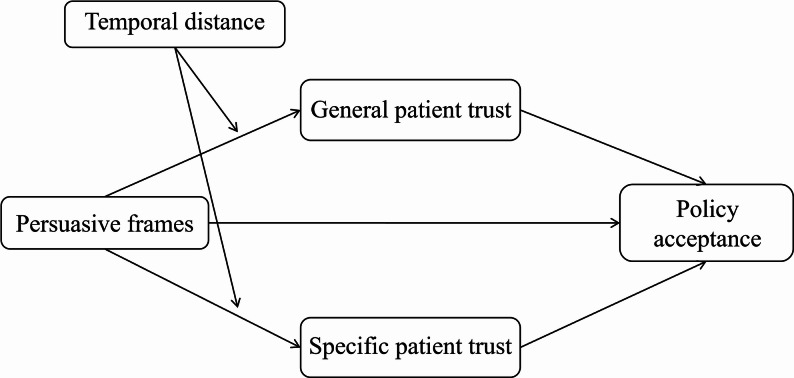
Conceptual framework

The study’s overall conceptual framework is illustrated in Fig. 1.

## Method

### Procedures and participants

Upon IRB approval and permission from the hospital, a survey experiment was conducted between January 11 and February 8, 2025, at a major maternal and child health hospital in Wuxi, a city in eastern China that serves as a national pilot site for DRG implementation. DRG implementation in obstetrics and gynecology has been proven effective with significantly reduced inpatient costs, shorter hospital stays, increased rates of minimally invasive surgery, and higher Case Mix Index (CMI) scores across China [53–55].

Only participants with no prior knowledge of DRGs were included. With the assistance of doctors and midwives, 233 questionnaires were distributed. Due to three reverse-scored items in the specific patient trust measure, questionnaires with identical responses to all items were deemed invalid, leaving a final sample of 229.

Participants were randomly assigned to either the personal interest frame (*n* = 115) or the social responsibility frame (*n* = 114). Age, gender, educational attainment, self-reported health status, and type of health insurance are key factors influencing patient trust [21, 27, 28]. Three rounds of independent sampling were conducted to control demographic differences across conditions.

To verify successful random assignment, a series of Chi-square tests were performed to assess demographic differences across conditions (Table 1), revealing no significant between-group differences.

**Table 1:** Sample demographics and differences in participants’ demographic characteristics in experimental conditions

Variables	Sample percentage	Test for differences by condition
Age		*p* = .300
18–29	28.8%	
30–39	48.5%	
40–49	17.0%	
50-	5.7%	
Education		*p* = .148
Less than primary school	4.4%	
Junior high diploma	6.1%	
High school diploma or some college degree	33.6%	
4-year college degree or above	59.8%	
Health insurance type		*p* = .917
No insurance	1.3%	
Employment-based insurance	66.8%	
Urban resident insurance	27.9%	
Rural resident insurance	3.9%	
Self-rated health status		*p* = .138
Very poor	4.4%	
Poor	2.2%	
Fair	33.2%	
Good	42.4%	
Very good	21.8%	

The stimulus materials were adapted from official government communications and publicly available media coverage regarding DRG reform, tailored to reflect two distinct framing conditions. Both versions provided an overview of DRG principles and reform objectives, including their relevance to obstetric care, supported by relevant data. The personal interest frame emphasized the reduction in personal medical expenses, hospital stays, and individual burden, along with more convenient reimbursement. The social responsibility frame highlighted controlling healthcare cost growth, preserving the healthcare fund, improving fund efficiency, and standardizing hospital practices. The full texts of both framing messages are provided in the supplementary material.

### Measurements

Framing condition was treated as a binary independent variable, coded as 0 = personal interest frame and 1 = social responsibility frame.

Childbirth constitutes the inpatient event with the shortest psychological distance for pregnant women. Accordingly, temporal distance was operationalized as the number of days remaining until the expected due date. It is important to note that DRG payment reform applies exclusively to inpatient services (e.g., childbirth and surgery) and does not cover outpatient services such as routine prenatal examinations; this distinction was explicitly stated in both experimental materials. Because most obstetric inpatients are admitted close to their due dates, the distribution of temporal distance was positively skewed (M = 4.92 weeks, SD = 7.56, range = 1–32 weeks). To address this skewness and facilitate analysis, temporal distance was categorized into four groups: within 1 week, within 1 month, within 10 weeks, and more than 10 weeks before the due date, coded from 1 to 4. Nearly half of the participants were within one week of their expected due date, whereas the remaining three categories were relatively evenly distributed.

Patient trust can be divided into general trust in the healthcare system and the medical profession, and specific trust in individual physicians [32]. General patient trust was measured by two items: “I trust the healthcare system in China” (Q3) and “I trust the medical profession” (Q4), rated on a 5-point Likert scale (1 = strongly disagree; 5 = strongly agree) (M = 3.88, SD = 0.44, α = 0.80). Specific patient trust was assessed using the Chinese version of the Wake Forest Physician Trust Scale (WFPTS-C-10) [56, 57], which includes 10 items (L1–L10) rated on a 5-point scale (1 = strongly disagree; 5 = strongly agree), with three items reverse-scored. Participants evaluated their attending physician, with higher scores indicating greater trust (M = 3.89, SD = 0.30, α = 0.89).

Policy acceptance was measured using a single-item indicator on a 5-point Likert scale (1 = strongly disagree, 5 = strongly agree), which assessed respondents’ support for the government’s decision to implement DRG-based payment reform. Single-item measures have demonstrated acceptable reliability and validity for concrete constructs in survey research, and prior studies have used single questions to assess policy acceptance [36]. Nevertheless, we acknowledge that single-item measures may not fully capture the multidimensional nature of this construct.

Data analysis was conducted using R (version 4.4.3).

## Results

**Fig. 2:**
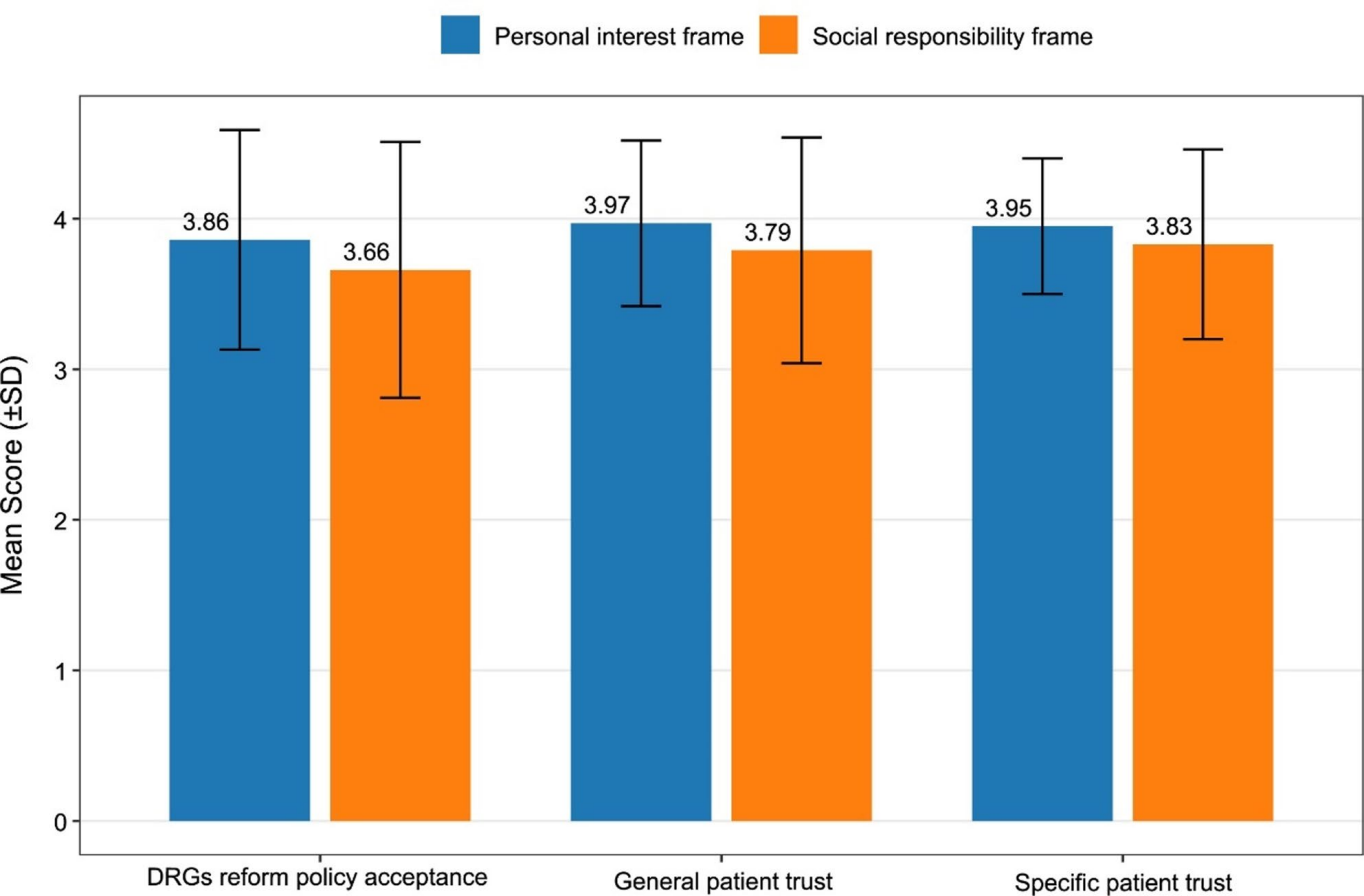
Dependent variables across frames

Overall, participants exhibited moderately high levels of patient trust and policy acceptance, as shown in Fig. 2. The experimental group exposed to the personal interest frame reported higher scores on all dependent variables compared to the group exposed to the social responsibility frame.

Independent samples t-tests were conducted to examine H1 (Table 2). The results indicated that H1a was supported (t = 2.067, *p* = .040), revealing a significant difference in general patient trust between the framing conditions. Specifically, the difference was significant for trust in the healthcare system (t = 1.977, *p* = .049), whereas trust in the medical profession did not reach statistical significance (t = 1.792, *p* = .075). H1b and H1c were not supported, as no significant differences were observed between conditions in specific patient trust or acceptance of the DRG reform policy.

**Table 2:** Group differences in general patient trust, specific patient trust and policy acceptance

Variables	M(SD)	Personal interest frame M (SD)	Social responsibility frame M(SD)	Test for differences by condition
General patient trust	3.88(0.66)	3.97(0.55)	3.79(0.75)	t = 2.067, *p* = .040*, Cohen’s d = 0.27
Specific patient trust	3.89(0.55)	3.95(0.45)	3.83(0.63)	t = 1.721, *p* = .086, Cohen’s d = 0.23
Policy acceptance	3.76(0.80)	3.86(0.73)	3.66(0.85)	t = 1.837, *p* = .067, Cohen’s d = 0.24

Note: *p < .05; **p < .01; ***p < .001

H2 was supported. Linear regression analysis revealed that general patient trust was positively associated with specific patient trust (β = 0.453, *p* < .001), after controlling for age, education level, insurance status, and self-reported health (Table 3). This suggests that higher levels of general trust are linked to higher levels of specific trust. Age was negatively associated with specific patient trust, whereas education level showed a positive association. Insurance status and self-reported health were not significantly related to specific patient trust. The overall model was significant (*p* < .001) and explained 39.87% of the variance in specific patient trust.

**Table 3:** Regression model predicting specific patient trust

Variable	Coefficient	Standard Error
General Patient Trust	0.453***	0.043
Age	-0.123***	0.035
Education Level	0.107*	0.046
Health Insurance Status	-0.027	0.051
Self-Reported Health Status	0.029	0.036
Constant	1.947***	0.312
Observations	229	
R^2^	0.399	
Adjusted R^2^	0.385	
Residual Standard Error	0.429 (df = 223)	
F Statistic	29.573*** (df = 5; 223)	

Note: *p < .05; **p < .01; ***p < .001

To examine whether temporal distance moderated the paths of the mediation model, we conducted a moderated mediation analysis using structural equation modeling in R (lavaan package) with 5,000 bootstrap resamples (Table 4). All variables were treated as observed variables.

The results supported H3a. Framing significantly predicted general patient trust (β = −0.144, *p* = .026), which in turn significantly predicted policy acceptance (β = 0.285, *p* = .004). The indirect effect of framing on policy acceptance through general patient trust was significant (β = − 0.041, 95% bootstrap CI [-0.145, -0.003]), as the confidence interval did not include zero. H3b was not supported. Framing did not significantly predict specific patient trust (β = −0.110, *p* = .091), and specific patient trust was not significantly associated with policy acceptance (β = 0.124, *p* = .156). Accordingly, the indirect effect of framing on policy acceptance via specific patient trust was not significant (β = − 0.014, 95% bootstrap CI [− 0.069, 0.013]), as the confidence interval included zero. The total indirect effect of framing on policy acceptance was significant (β = −0.055, *p* = .032, 95% bootstrap CI [− 0.167, − 0.008]), while the direct effect of framing on policy acceptance was not significant (β = −0.077, *p* = .235). Together, these findings indicate that the influence of framing on policy acceptance operates primarily through indirect pathways, particularly via general patient trust.

To further examine the robustness of the proposed mediation structure, reverse mediation models were tested in which policy acceptance was specified as the mediator between framing and the two trust variables. The results showed that policy acceptance did not mediate the effect of framing on general patient trust (indirect effect = − 0.057, 95% bootstrap CI [− 0.124, 0.000]) or specific patient trust (indirect effect = − 0.037, 95% bootstrap CI [− 0.082, 0.000]), as the confidence intervals included zero.

**Table 4:** Mediation analysis of the effects of framing on policy acceptance via general patient trust and specific patient trust

Mediation Path	β	*p*	95% Confidence Interval (CI)
Hypothesis 3a (H3a): Framing → General Patient Trust → Policy Acceptance			
Framing → General Patient Trust	-0.144	0.026*	[-0.353, -0.022]
General Patient Trust → Policy Acceptance	0.285	0.004**	[0.093, 0.555]
Indirect Effect (Framing → General Patient Trust → Policy Acceptance)	-0.041	0.049*	[-0.145, -0.003]
Hypothesis 3b (H3b): Framing → Specific Patient Trust → Policy Acceptance			
Framing → Specific Patient Trust	-0.110	0.091	[-0.258, 0.017]
Specific Patient Trust → Policy Acceptance	0.124	0.156	[-0.071, 0.426]
Indirect Effect (Framing → Specific Patient Trust → Policy Acceptance)	-0.014	0.296	[− 0.069, 0.013]

Note: *p < .05; **p < .01; ***p < .001

H4 was not supported. Temporal distance did not significantly predict any of the dependent variables. Specifically, it did not significantly predict policy acceptance (β = 0.087, *p* = .141), general patient trust (β = 0.131, *p* = .064), or specific patient trust (β = -0.010, *p* = .886). Furthermore, the interaction between framing and temporal distance did not significantly predict general patient trust (β = -0.042, *p* = .660) or specific patient trust (β = -0.063, *p* = .512). These results indicate that temporal distance did not moderate the effects of framing on either mediator.

## Discussions and conclusion

### Discussions

While prior research on DRG-based payment reform has primarily concentrated on cost containment and provider behavior, this study shifts the analytical focus toward policy communication and its trust mechanism. The personal interest frame was found to be more effective than the social responsibility frame in enhancing patients’ general trust, with the difference driven primarily by trust in the healthcare system rather than trust in the medical profession. This suggests that policy communication should prioritize aligning messages with patients’ immediate interests rather than merely emphasizing social benefits. Although the observed effect sizes were limited, such magnitudes are consistent with prior framing research, given that exposure to a single piece of policy information is unlikely to produce large attitudinal shifts.

However, exposure to different frames did not produce a direct effect on patients’ trust in specific healthcare providers or the medical professional. Also, general patient trust was found to mediate the relationship between framing and policy acceptance, whereas specific patient trust did not. This asymmetric pattern of effects provides further empirical support for the analytical distinction between general and specific trust. General patient trust, which reflects evaluations of the healthcare system and institutional arrangements, appears more cognitively responsive to policy-related information. In contrast, trust in specific healthcare providers or the medical profession is likely shaped by accumulated interpersonal experiences, professional norms, and long-term interactions, rendering it less susceptible to short-term informational framing.

Consistent with prior research, general patient trust was positively associated with specific patient trust, indicating a hierarchical trust structure in which confidence in the healthcare environment may spill over into evaluations of individual providers. Additionally, age was found to be negatively related to specific patient trust, while education level was positively related. This suggests that health policy communication might be tailored to different groups. Beyond improving interpersonal communication, efforts should also focus on strengthening the healthcare system and ensuring that policy information is communicated in a clear and accessible manner.

Meanwhile, temporal distance and its interaction with framing showed no significant effects, likely due to several methodological and substantive factors. The operationalization of temporal distance through expected delivery timing may not fully capture participants’ perceived psychological distance from the DRG policy. Although the experimental materials specified that DRG applies only to inpatient services, participants’ actual understanding of the policy was not directly measured. Respondents thus may not have perceived DRG as temporally or situationally distant, but rather as part of a continuous and pervasive medical environment. Moreover, pregnant women typically experience a continuous and intensive engagement with the healthcare system across both prenatal and postnatal stages. Their perceived hypothetical distance to healthcare policies may be relatively homogeneous, limiting the extent to which temporal distance differentiates psychological distance. On the other side, trust in medical institutions and professionals is often shaped through long-term experiences and socialization, making it a relatively stable construct that is less sensitive to short-term contextual cues such as perceived timing. In contrast, temporal distance is more situational and fluid, and may therefore be insufficient to alter entrenched trust judgments. Future studies could employ more refined measures of psychological distance and investigate its moderating role in populations with more diverse care trajectories.

Taken together, the findings suggest that policy communication frames may shape public trust toward the healthcare system even when their direct effects on policy acceptance are limited. In turn, trust in the healthcare system may influence attitudes toward reform policies. To situate these findings, it is useful to consider the broader policy context. China’s policy process is widely characterized by strong hierarchical steering and centralized agenda-setting under a “top-level design” approach [58], within which public participation is often regarded as costly, informationally inefficient, or potentially disruptive [59]. Such concerns are particularly salient in the field of public health, where highly specialized knowledge is often deemed indispensable, giving rise to a long-standing “government–scientist” dual decision-making model [10].

DRG reform thus represents a contested domain in which an expert-driven model may enhance short-term efficiency but also raises questions about long-term legitimacy when public deliberation is constrained. Although the present study does not directly assess long-term legitimacy outcomes, the findings show that even modest variations in framing can shape general trust in the healthcare system under controlled experimental conditions. In this sense, policy communication emerges as an important yet often underexamined link between expert-driven policy design and public legitimacy in China’s healthcare reform.

### Limitations and further research

The study has several limitations. First, data collection was conducted with the assistance of physicians or midwives, which may have introduced social desirability bias and systematically suppressed the expression of negative evaluations toward the medical system or related policies [60]. To mitigate this concern, participation was voluntary, questionnaires were completed anonymously, and medical staff were not present during the survey completion. Nevertheless, the involvement of medical authorities at the recruitment stage may still have influenced participants’ responses, potentially biasing trust-related measures upward and affecting the internal validity of the findings.

Second, the sample was drawn from obstetric patients in a single hospital, which may constrain external validity. Obstetric care involves relatively standardized inpatient procedures and clear insurance reimbursement pathways, which may shape patients’ perceptions of DRG reforms differently compared to patients in departments with more complex or discretionary treatment decisions. Moreover, pregnant women face a relatively fixed and imminent inpatient event, which may amplify the salience of personal cost considerations. Patients with chronic conditions or acute but unpredictable illnesses may process policy information differently, and framing effects may therefore vary across clinical contexts.

Third, regarding measurement design, this study relies on a single-item measure of policy acceptance. While such measures may be suitable for simple constructs, policy acceptance is inherently multidimensional, encompassing aspects such as policy support and personal acceptance [61], and thus cannot be adequately captured by a simple dichotomy of “accept” versus “not accept.” This limitation introduces greater measurement error, which may attenuate relationships in the mediation model; consequently, it also prevents us from distinguishing which specific dimension of policy acceptance is influenced by the mediators. Regarding the measurement of specific patient trust, although the scale demonstrated high reliability, it was translated and included reverse-coded items; given the cognitive load inherent in medical settings, some participants may have misinterpreted certain items, thereby introducing additional measurement error.

Fourth, the study did not include a manipulation check to verify whether participants perceived the framing conditions as intended. The absence of such a check limits confidence in the interpretation of both significant and null effects, as it remains unclear whether the observed results reflect the true absence of framing effects or insufficient differentiation between experimental conditions. Furthermore, due to the institutional complexity of the DRG policy, the experimental materials necessarily simplified policy details, which may have limited participants’ understanding and reduced the ecological validity of the stimuli. Relatedly, the two framing conditions differed in both the level and type of information presented, with the personal interest frame relying on concrete, patient-level figures and the social responsibility frame emphasizing more aggregate, abstract system-level indicators; this difference may have influenced information processing and perceived relevance, thereby complicating the attribution of observed effects solely to framing orientation.

Future research could extend the present study in several directions. During participant recruitment, a pronounced asymmetry in policy awareness was observed, as physicians were consistently familiar with the DRG policy, whereas very few patients reported prior knowledge of it. This suggests the need for systematic investigation into how gaps in policy awareness beyond medical expertise shape patients’ trust formation, policy acceptance, and responses to policy communication within expert-driven governance contexts. Moreover, the framing effects identified in this study may vary across patient populations and clinical settings. Patients with chronic conditions, acute but unpredictable illnesses, or those treated in different institutional environments may process policy information differently, as their healthcare experiences, uncertainty levels, and dependency on the system vary. Finally, while the current study captures immediate attitudinal responses to policy framing, trust in healthcare institutions is often formed and adjusted over time through accumulated experiences. Future studies could therefore adopt longitudinal or mixed-method approaches to examine how short-term framing effects interact with longer-term experiences under DRG-based payment systems.

### Conclusion

Framing policies in terms of personal benefits was associated with higher levels of general patient trust compared to emphasizing social responsibility. Although the framing manipulation did not produce a direct difference in policy acceptance, general patient trust emerged as a significant psychological pathway linking message framing to policy acceptance and was significantly associated with specific patient trust. These findings highlight the importance of clear and accessible policy communication in healthcare reform, particularly in contexts where public awareness remains limited. By shifting the focus from provider-centered cost control to patient-centered communication and trust-building, this study adds a valuable perspective to ongoing discussions of DRG payment reform.

## Electronic supplementary material

Below is the link to the electronic supplementary material.

Supplementary Material 1

## Data Availability

The datasets used and/or analysed during the current study are available from the corresponding author on reasonable request.
