# Supplementary material for: Trust by design: the effects of personal interest vs. social responsibility frame on policy acceptance and patient trust in China’s DRG reform
Source: BMC Health Serv Res. 2026 May 11;26:917. doi: 10.1186/s12913-026-14681-1 (PMC13339517; doi:10.1186/s12913-026-14681-1)
Supplement: Supplementary file 1 — Supplementary Material 1 [file 12913_2026_14681_MOESM1_ESM.pdf]

The stimulus materials were adapted from official government communications and publicly available media coverage regarding DRG reform, tailored to reflect two distinct framing conditions. Both versions provided an overview of DRG principles and reform objectives, including their relevance to obstetric care, supported by relevant data. The original material is in Chinese, and the English version is provided below.

Personal interest frame

Please carefully read the following introduction to the reform of the DRG-based medical insurance payment system.

Diagnosis Related Groups (DRGs) are a case-mix classification scheme. After the implementation of DRG-based medical insurance payment, some regions have seen a significant reduction in average medical expenses per patient, length of hospital stay, and individual financial burden, while reimbursement and settlement procedures have become more convenient. Taking Wuxi as an example, since the implementation of the DRG payment reform, the average actual medical expenses in pilot hospitals decreased by 916.31 yuan in 2020 compared with the previous year, and further declined by 839.1 yuan year-on-year from January to September 2021.

The concept of DRGs: Under the DRG payment system, each case is assigned to a specific diagnosis-related group based on differences in diagnosis, treatment methods, and patient characteristics. For patients within the same group, similar cost standards are determined, and the medical insurance authority provides a “bundled” payment to medical institutions. If the hospital’s actual cost of treating a given disease is lower than the DRG payment, the saved amount becomes hospital revenue; if the cost exceeds the payment, the hospital bears the excess itself. This fundamentally changes the incentive mechanism for medical institutions and healthcare professionals, shifting from “earning revenue by providing more services” to “earning revenue by controlling costs.”

Wuxi is the first city in Jiangsu Province to become a national demonstration site for DRG payment. At present, 189 medical institutions providing inpatient services have implemented actual DRG-based payments. DRG-based medical insurance payment applies only to inpatient cases requiring hospitalization, such as pregnancy and childbirth-related surgical procedures, and does not apply to outpatient cases such as prenatal examinations.

Social responsibility frame

Please carefully read the following introduction to the reform of the DRG-based medical insurance payment system.

Diagnosis Related Groups (DRGs) are a case-mix classification scheme. Implementing DRG-based medical insurance payment reform can curb the unreasonable growth of medical expenses, preserve the medical insurance fund as a financial reservoir, and improve the efficiency of fund utilization. From the hospital perspective, clinical practices become more standardized, and medical expenditures receive reasonable compensation. In some reform pilot cities, medical insurance funds have achieved a balance between income and expenditure, ensuring that patients who do not require hospitalization are no longer admitted at a low standard, while those who truly need hospitalization are able to be admitted. In 2021, two-thirds of the pilot hospitals in Wuxi achieved a surplus, with a total

retained surplus of 224 million yuan. The overall payment rate of employee medical insurance increased by 2.12 percentage points compared with the previous year, while the payment rate of resident medical insurance increased by 12.73 percentage points.

The concept of DRGs: Under the DRG payment system, each case is assigned to a specific diagnosis-related group based on differences in diagnosis, treatment methods, and patient characteristics. For patients within the same group, similar cost standards are determined, and the medical insurance authority provides a “bundled” payment to medical institutions. If the hospital’s actual cost of treating a given disease is lower than the DRG payment, the saved amount becomes hospital revenue; if the cost exceeds the payment, the hospital bears the excess itself. This fundamentally changes the incentive mechanism for medical institutions and healthcare professionals, shifting from “earning revenue by providing more services” to “earning revenue by controlling costs.”

Wuxi is the first city in Jiangsu Province to become a national demonstration site for DRG payment. At present, 189 medical institutions providing inpatient services have implemented actual DRG-based payments. DRG-based medical insurance payment applies only to inpatient cases requiring hospitalization, such as pregnancy and childbirth-related surgical procedures, and does not apply to outpatient cases such as prenatal examinations.
